# Challenges Facing First-Generation College Graduates in Medical School: A Qualitative Analysis

**DOI:** 10.1001/jamanetworkopen.2023.47528

**Published:** 2023-12-13

**Authors:** Catherine Havemann, Hyacinth R. C. Mason, Regina G. Russell, Alejandra Casillas, Mytien Nguyen, Dowin Boatright, Alexis Webber, Jon Andre Parrilla, Abraham Gallegos, Tasha R. Wyatt

**Affiliations:** 1 Section of Emergency Medicine, University of Chicago, Chicago, Illinois; 2 Tufts University School of Medicine, Boston, Massachusetts; 3 Vanderbilt University School of Medicine, Nashville, Tennessee; 4 Division of General Internal Medicine and Health Services Research, Department of Medicine, UCLA David Geffen School of Medicine, Los Angeles, California; 5 Yale School of Medicine, New Haven, Connecticut; 6 Ronald O. Perelman Department of Emergency Medicine, Emergency Medicine and Population Health, NYU Grossman School of Medicine, New York, New York; 7 Department of General Surgery, Albany Medical Center, Albany, New York; 8 Kirk Kerkorian School of Medicine at University of Nevada Las Vegas; 9 Kaiser Permanente Bernard J Tyson School of Medicine, Pasadena, California; 10 Center for Health Professions Education, Uniformed Services University, Bethesda, Maryland

## Abstract

**Importance:**

First-generation (FG) medical students remain underrepresented in medicine despite ongoing national efforts to increase diversity; understanding the challenges faced by this student population is essential to building holistic policies, practices, and learning environments that promote professional actualization. Although FG students have been extensively studied in the undergraduate literature, there is little research investigating how FG students experience medical education or opportunities for educators to intervene.

**Objective:**

To explore challenges that FG students experience in undergraduate medical education and identify opportunities to improve foundational FG support.

**Design, Setting, and Participants:**

This qualitative study was conducted using an online platform with 37 FG students enrolled in 27 US medical schools. An interprofessional team of medical educators and trainees conducted semistructured interviews from November 2021 through April 2022. Participants were recruited using a medical student listserv. Data were analyzed from April to November 2022.

**Main Outcomes and Measures:**

After conducting a preliminary analysis using open coding, a codebook was created and used in a thematic analysis; the codebook used a combination of deductive and inductive coding.

**Results:**

Among the 37 students recruited for this study, 21 (56.8%) were female; 23 (62.2%) were in the clinical phase of training; 1 (2.7%) was American Indian or Alaska Native, 7 (18.9%) were Hispanic, Latino, or of Spanish origin, 8 (21.6%) were non-Hispanic Asian or Asian American, 9 (24.3%) were non-Hispanic Black or African American, and 23 (32.4%) were non-Hispanic White; mean (SD) age was 27.3 (2.8) years. Participants described 4 major themes: (1) isolation and exclusion related to being a newcomer to medicine; (2) difficulty with access to basic resources (eg, food, rent, transportation) as well as educational (eg, books); (3) overall lack of faculty or institutional support to address these challenges; and (4) a sense of needing to rely on grit and resilience to survive.

**Conclusions and Relevance:**

Although grit and resilience are desirable traits, results of this study suggest that FG medical students face increased adversity with inadequate institutional support, which forces them to excessively rely on grit and resilience as survival (rather than educational) strategies. By applying the holistic model often used in admissions to the postmatriculation educational process, targeted and flexible initiatives can be created for FG students so that all students, regardless of background, can achieve robust professional actualization.

## Key Points

**Question:**

What challenges do first-generation college graduates face in medical school, and how can their schools better support them?

**Findings:**

This qualitative study of 37 first-generation college graduates in medical school found that participants described 4 major themes: isolation and exclusion, challenges with access to resources, lack of institutional support, and need to self-rely on grit and resilience to survive.

**Meaning:**

Results from first-generation medical student narratives suggest that they perceive disproportionate adversity and insufficient institutional support in an educational phase already characterized by substantial challenges, leading to overreliance on grit and resilience as survival strategies.

## Teaser

This qualitative study explores challenges that first-generation students experience in medical education and identifies opportunities to improve foundational first-generation support.

## Introduction

As medicine recognizes the key role of diversity in addressing health equity,^1,2,3,4^ first-generation (FG) medical students have come into focus. FG students are students whose parents have not earned bachelor’s degrees.^5,6^ They tend to be older, from traditionally marginalized racial and ethnic groups, children of immigrants or are immigrants themselves, and/or come from families with low income.^7,8^ FG students also tend to be underrepresented in medicine, identifying as Black, Latinx, or Indigenous.^9,10^ Presently, between 12.4%^11^ and 14% of students^12^ enrolled in US medical schools are FG, and research suggests that they tend to struggle in unique ways compared with their peers.^10,11,12,13,14,15^ These struggles have led to an academic reputation for possessing “grit,”^16,17^ a quality commonly equated with self-reliance and understood as “perseverance and passion for long-term goals.”^18^

Despite their resilience, FG students’ experiences of ongoing struggle raise the question of how gritty they are expected to be as they pursue training. How much support should a student receive, and how much should they be able to rely on themselves to get through medical education? This study explores the challenges FG students experience in medical education and identifies opportunities for innovation where medical schools and educators can offer increased support.

## Methods

This qualitative study was approved by the Yale institutional review board, and participants provided electronic informed consent and permission to use their deidentified quotes for research purposes. We followed the Standards for Reporting Qualitative Research (SRQR) reporting guideline.

This study explored the experiences of FG medical students’ challenges in medical school between November 2021 and April 2022. It is part of a larger investigation into the professional identities of FG students and how coming from families without advanced educational and financial attainment influences their growing professional identities. This work was guided by the conceptual framework and values of holistic review, which is defined by the Association of American Medical Colleges (AAMC) as a balanced process of assessing applicants that considers their “experiences, attributes, and academic metrics, and…how the individual might contribute value as a student and physician.”^19^ Holistic review further “recognizes that diversity is critical to excellence,”^19^ and should ideally extend beyond admissions into “curriculum, student support, and faculty development.”^19^

We developed interview questions using published literature on FG students’ experiences in undergraduate education and the team members’ personal and professional knowledge of FG medical students. Questions pertained to factors affecting participants’ professional identities, including pre–medical school experiences, assets and challenges experienced in medical school, the school’s culture, sense of belonging, and participants’ perspectives on how to address some common challenges. We then piloted the interview questions with 3 FG graduates and revised the interview questions (eAppendix in Supplement 1) based on the pilot data.

We recruited medical students from FG family backgrounds via a listserv dedicated to the FG student community. Students were eligible for the study if they met the Higher Education Act of 1965 and 1998 definition of FG^5^ with neither parent and/or guardian having attained a bachelor’s degree. Interested participants completed an online screening tool that collected demographic information, which was used to purposefully sample a diverse group of FG medical students. Race and ethnicity data were self-reported.

Interviews lasted 25 to 65 minutes and were recorded over Zoom. In line with constructivist grounded theory^20^ approaches to developing interview questions, we iteratively revised our questions as needed after each interview. Given how diverse FG students are, and how varied their answers were, we adjusted our questions to capture participants’ experiences. Each participant received a $20 gift card for participation and recruitment ceased when we reached thematic sufficiency.

### Data Analysis

We began formal data analysis after reviewing the first 4 interviews and open coding for insights into FG students’ experiences. After multiple discussions about the codes, we created a codebook and used Dedoose version 9.017 (SocioCultural Research Consultants) for analysis. All transcripts were coded at least twice, and disagreements were resolved through discussion. The team met regularly to explore and challenge emerging ideas, develop intersubjectivity, strengthen arguments, resolve differences, and explore “aspects of the inquiry that might otherwise remain only implicit within the inquirer’s mind.”^21^ Following this, we used a combination of an inductive and deductive thematic analysis to understand the relationship between the codes. Once data analysis was complete, we conducted member checking with 3 participants; results supported our interpretation.

The team included 4 medical trainees and 6 faculty members, most of whom were FG college graduates themselves. Some team members hold formal institutional positions supporting FG students. Additionally, trainee members of the study team had experienced many of the same issues as medical students. Each team member thus brought professional and personal expertise to the analytical sense-making process. We consistently engaged in cross-examination for similarities and differences between our experiences and what students described to ensure that our interpretations were grounded in the data students provided. Data analysis was performed from April to November 2022.

## Results

Among the 37 students from 27 medical schools recruited for this study, 21 (56.8%) were female; 23 (62.2%) were in the clinical phase of training; 1 (2.7%) was American Indian or Alaska Native, 7 (18.9%) were Hispanic, Latino, or of Spanish origin, 8 (21.6%) were non-Hispanic Asian or Asian American, 9 (24.3%) were non-Hispanic Black or African American, and 23 (32.4%) were non-Hispanic White; 17 (46.0%) were from groups underrepresented in medicine (American Indian or Alaska Native; Hispanic, Latino, or of Spanish origin; non-Hispanic Black or African American); mean (SD) age was 27.3 (2.8) years, and 28 participants (75.6%) were aged 23 to 29 years. The Table displays characteristics of the participants.

**Table.** Participant Demographic Characteristics Among FG Medical Students Who Completed Interviews About Professional Identity Formation

Characteristics	FG only (n = 6)	FG/LI (n = 31)	Total (FG and FG/LI) (N = 37)
Sex			
Male	3 (50.0)	13 (41.9)	16 (43.2)
Female	3 (50.0)	18 (58.1)	21 (56.)
Race and ethnicity			
American Indian or Alaska Native	0	1 (3.2)	1 (2.7)
Hispanic, Latino, or of Spanish origin	1 (16.7)	6 (19.4)	7 (18.9)
Non-Hispanic Asian or Asian American	1 (16.7)	7 (22.6)	8 (21.6)
Non-Hispanic Black or African American	2 (33.3)	7 (22.6)	9 (24.3)
Non-Hispanic White	2 (33.3)	10 (32.3)	12 (32.4)
Generation status			
FG only	6 (100.0)	0	6 (16.2)
FG/LI	0	31 (100.0)	31 (83.8)
Highest level of education completed by primary parents/parental figures			
Elementary	0	4 (12.9)	4 (10.8)
Some high school	0	1 (3.2)	1 (2.7)
High school graduate/GED	2 (33.3)	21 (67.7)	23 (62.2)
Associate degree or equivalent	2 (33.3)	2 (6.5)	4 (10.8)
Some college, no bachelor’s degree	1 (16.7)	3 (9.7)	4 (10.8)
Bachelor’s degree	0	0	0
NA	1 (16.7)	0	1 (2.7)
Program type			
MD	6 (100.0)	29 (93.5)	35 (94.6)
DO	0	2 (6.5)	2 (5.4)
Phase of school			
Clinical	3 (50.0)	20 (64.5)	23 (62.2)
Preclinical	3 (50.0)	11 (35.5)	14 (37.8)
Age, y			
23-25	4 (66.6)	9 (29.0)	13 (35.1)
26-29	1 (16.7)	14 (45.3)	15 (40.5)
≥30	1 (16.7)	8 (25.8)	9 (24.3)
Family income growing up, $			
0-49 999	0	25 (80.7)	25 (67.6)
50 000-119 999	4 (66.7)	6 (19.3)	10 (27)
120 000-199 999	1 (16.7)	0	1 (2.7)
Do not know	1 (16.7)	0	1 (2.7)
Prefer not to answer	0	0	0
SNAP or federal assistance programs growing up			
Yes	1 (16.7)	17 (54.8)	18 (48.6)
No	5 (83.3)	10 (32.3)	15 (40.5)
Do not know	0	3 (9.7)	3 (8.1)
Prefer not to answer	0	1 (3.2)	1 (2.7)

Abbreviations: DO, doctor of osteopathic medicine; FG, first-generation; FG/LI, first-generation and low-income; GED, General Educational Development; MD, doctor of medicine; NA, not applicable; SNAP, Supplemental Nutrition Assistance Program.

FG medical students indicated that they struggled in several key areas that made medical school particularly challenging. This included having fewer resources compared with their peers; inadequate support from their institutions and faculty members; feeling othered or isolated within medical school; and needing constant resourcefulness and resilience to overcome challenges.

### Access to Resources

FG students experienced tremendous financial stress, which permeated every aspect of their medical training. Because many of them came from families with low income, they experienced numerous hardships trying to pay for mandatory expenses such as board examinations, study resources, away-rotation fees, residency application costs, and even basic needs such as food, rent, and transportation. They identified many hidden costs in medical school that were unexpected. For example, 1 participant explained that it is assumed or expected that students have access to personal transportation on clerkships, and that this can even be a formal obligation from medical schools: “When we matriculate, we sign a contract saying we will buy a car. I can’t buy a car and live in [city]!” (participant 30).

As another student explained that not having the assumed resources was isolating because their peers seemed to not struggle in the same ways. Participants perceived non-FG students as easily able to afford needed resources: “I felt like they don’t understand. They worry about school. I’m also worrying about whether I’m going to eat, have money for rent, or afford paying for this mandatory examination that is $645” (participant 5).

This persistent and consistent lack of resources influenced FG students’ ability to learn. Without the extra support of tutors and expensive extracurricular resources, they often felt that they required additional time and effort to catch up with their peers. Due to ubiquitous use, participants noted that extra study resources are essentially required and the inability to afford them takes an academic toll.

“I’ve seen how money can buy resources that you need to do well in school. When I meet my peers, they’ve so much additional support in place. I study 10 hours to learn something compared to others who study for half that time” (participant 17).

Financial stress forced many students to seek sources of income such as substitute teaching, selling goods online, recycling, or working retail or in construction. The need to for survival income came at a cost as well: “Instead of focusing on studying or research, I’m spending 4 hours at the plasma clinic to get $100” (participant 31).

### Lack of Institutional Support

Participants noted that their institutions often praised FG students’ engagement and contributions to the school but failed to recognize that these efforts reflect unmet needs for which institutional support was more appropriate. As 1 participant explained, “[They] compliment us, [by saying] ‘Our students take charge, they’re amazing!’ [And, I want to say] ‘Hey! You think you can support us, maybe?’” (participant 14). Overall, FG students felt they did not receive the level of institutional support that they needed, which included career advice, educational and psychological support, and disability accommodations. At times the lack of support even took the form of active discouragement, such as mentors who advised FG students against competitive specialties. One student (who later matched into dermatology) shared: “I voiced concern to my dean that certain specialty mentors couldn’t give me [career] advice since we were from such different backgrounds. The response was, ‘…maybe the culture of dermatology isn’t for you, you should drop pursuing [it].’ [They] said that I should delay graduation, not go through the Match and find other things I could do with an MD” (participant 25).

Another student was told repeatedly by advisers and evaluators that they should not become a physician because of their familial circumstances: “I’ve been told, ‘Maybe you shouldn’t be a doctor…medicine’s not for you because your parents aren’t doctors or high-ranking’” (participant 27).

Some participants felt a lack of support because they were not only FG, but from a racially marginalized community. One student explained, “I’m Native [American]. For the first time, I’m in a place where there isn’t a good support system for people like me. I felt alone, not adequately supported, and not understood. That was really hard” (participant 17).

The more intersectional a student’s identity, the harder it seemed to be. For example, a student talked about their experiences with a learning disability: “No one wondered whether I had a learning disability. What 28-year-old has an undiagnosed learning disability?...The problems I had [in medical school] were attributed to being Latino” (participant 20).

The cumulative effect of these experiences was the feeling that institutions had not concretely addressed pain points in the educational climate and could not adequately support FG students. For some, this resulted in leaves of absences to address school-related anxiety and depression: “I had a hard time transitioning into medical school. I didn’t feel supported from the start. I felt pressure to be successful…[and] they told me to take a leave of absence because of my depression” (participant 15).

### Isolation and Exclusion

FG students often felt excluded and isolated from their peers and faculty members because they found it difficult to connect without shared experiences and mutual understanding. “I’m cognizant that [medicine] doesn’t have a lot of people like me. [These] spaces that weren’t built for people like me and aren’t always welcoming. [I have] challenges I face that my peers can’t relate to or understand…it’s lonely” (participant 16).

Participants were overwhelmed by the new, often opaque pedagogies, language systems, and rules of conduct with which their peers seemed more comfortable: “There is a hidden curriculum. The way that things are taught or articulated in writing and verbally—the whole thing was completely new for me, relative to my peers” (participant 22). As a result, students described self-doubt regarding their overall belonging and worthiness in medicine: *“*Insecurity affected which careers seemed like options. [I] felt dumb for considering surgery. I just don’t belong.” (participant 30).

Students’ sense of isolation and exclusion was exacerbated by lack of resources: “There’s this low-dose anxiety that keeps following me as I go through this. It tells me, you can’t be like other people. They got the money, the time, and the resources. You don’t. You have to secure this for yourself” (participant 43). This was further compounded by feeling that they could not discuss money-related issues with faculty and administration: “I told them, ‘I know I do well when I do a ton of practice questions.’ Their suggestions [were] to use expensive secondary online resources. I didn’t feel comfortable telling them these aren’t practical options. It was like they didn’t really know who I was” (participant 23). Ultimately, lack of attention to the specific needs and financial constraints of FG students contributed to feelings of being outsiders in their own institutions, which affected their mental health through the isolating self-reliance required to meet their needs.

### Reliance on Resilience

Participants viewed themselves as having unique sociocultural assets that informed their self-concept as future physicians, offered benefits to their future patients, and helped them to overcome the multiple obstacles they experienced. These assets were drawn from personal experiences and contextualized by their backgrounds. Across a variety of these sociocultural contexts, participants spoke of resilience in the face of barriers: “During those 2 years, I had family things on my mind. MCAT [Medical College Admission Test], a full-time job, and applying to med school. I’m a pretty resilient person” (participant 13).

Participants argued for the value of their work ethic and ability to persist: “First-generation students make a way out of no way, figure out how to get things done, even without mentorship or advising” (participant 2). Some students were motivated to disprove negative stereotypes associated with their sociocultural background. ***“***There’s always something inside of me that I have to prove that I deserve to be here” (participant 34).

This ability to call on internal resources had a negative side, as many students expressed difficulty asking for academic help. “My ability to do [everything] for myself, reinforced the idea that I can’t ask for help or reach out to say, ‘Hey, I’m struggling’” (participant 43). Many felt they had to depend on individual strategies for academic and professional advancement because of the lack of support offered: “How would I have fared if I wasn’t as proactive? Would school have reached out? Helped me with resources? Would they have recommended I get tested? I wonder how a student who didn’t ask for help would fare” (participant 16).

## Discussion

Grit is understood as an individual’s commitment to “passion and perseverance for long-term goals,”^18^ which was clearly demonstrated in the participants’ narratives. Participants also found themselves engaged in ongoing acts of resilience as they continuously adapted to adversity and trauma.^22^ In medical education, these 2 constructs are associated with both personal and academic well-being^23^; however, our study strongly suggests that FG medical students are experiencing substantial challenges despite using grit and resilience to meet them. The presence of these characteristics raises the question: how gritty are FG students expected to be? Although grit and resilience are highly desirable traits in medical trainees,^23^ we posit that the ideal application of these qualities is toward the immutable challenges of medical training: the intricacies of pathophysiology, performing a hypothesis-driven history and physical examination, and bearing compassionate witness to human pain and hardships. In the current educational milieu, FG students are instead relying on grit and resilience to battle overwhelming financial stress, a labyrinthine or opaque educational system, and a hidden curriculum with implicit, often unwelcoming professional norms and expectations. Accordingly, we posit that the requirement to conform to the structures and norms of this system has forced an overreliance on these strategies, leaving FG students disproportionately stuck in survival mode with inadequate or imprecise institutional support. Ultimately, they are preempted from the opportunity to use their considerable assets toward professional actualization.

To meet the ideal of a diverse physician workforce, we must create learning environments where all students can thrive. FG students bring insights and experiences that are vital to meeting the needs of our health care system through their ability to see, connect with, and deeply understand our patients’ sociocultural diversity.^24,25^ Success for FG students requires educators to acknowledge that students exist in the full context of their background and their educational environment, which educators have enormous power to influence.^25^ Holistic strategies have helped medical schools make strides in more equitable admission policies and practices,^26^ but as these narratives indicate, admission is not the same as access. These narratives suggest that adversity, for FG students, does not end at matriculation, due in part to learning environments that are well-intended but fail to cultivate empowerment and achievement of diverse learners. By expanding our holistic lens, we can build student-centered frameworks such as the Integrated Holistic Student Affairs (IHSA) model. The IHSA model, developed by leadership of the AAMC’s Group on Student Affairs, provides a road map to shift “from reactive, deficit-oriented practices to proactive, empowering, equitable practices.”^27^ Accordingly, we suggest a holistic reimagining of merit,^28^ where merit encompasses not only apex academic achievement, but “distance traveled,” structural competency, advocacy, service, and mentorship. With these frameworks in mind, the Figure outlines strategies for educational equity derived from participant observations. Successful strategies will require creating a network of academic resources, reducing myriad financial barriers, and proactively creating space, visibility, and community for FG students.

**Figure.**
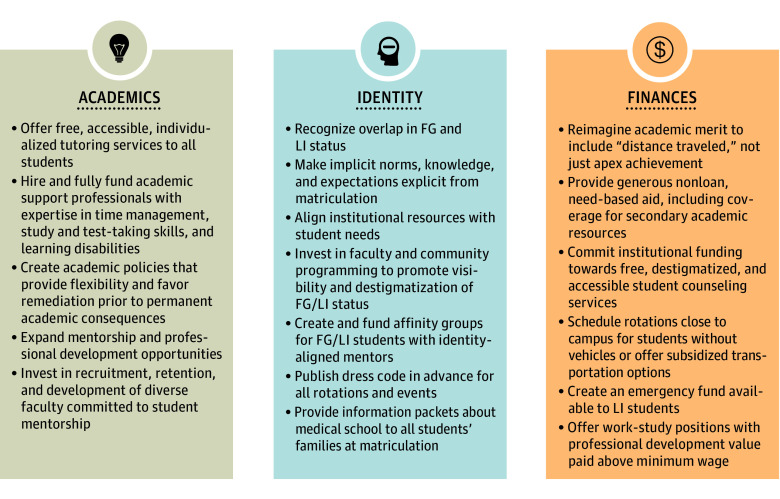
Support for First-Generation (FG) College Graduates in Medical School Targeted strategies for the support of FG college graduates in medical school. FG/LI indicates first-generation and/or low-income; LI, low-income.

Finally, many participants disclosed having a disability. Although the frequency of disability diagnosis among FG medical students is understudied, it is likely that FG students are uniquely affected by disability due to factors such as late diagnosis, lack of access to treatment, and overreliance on grit. Notably, students with disabilities as well as a high debt load experience even greater levels of stress,^29^ making it all the more critical to support this subpopulation. This is a rich area for future study as well as for targeted educational and programmatic interventions.

### Limitations

Our study has limitations. Because we included data from students who self-identified as FG, participants may not be a fully representative sample, and the results may not be generalizable to other groups, including students from low income–only backgrounds with parent(s) who have college degrees. We intentionally limited our study to focus on this specific population. Because FG students are often concurrently from families with low income, there are overlapping characteristics and future work should investigate FG-only compared with low income–only subgroups, and examine how students from poverty or low-income backgrounds experience medical education. Students who are FG-only but do not meet the definition of low income may still experience financial disparities due to the relative wealth of continuing-generation medical students. This merits further investigation. Our findings were hypothesis-generating; we are unable to make causal inferences.

## Conclusions

FG medical students represent a complex cohort whose assets and challenges merit innovation in educational frameworks. By shifting traditional conceptions of merit, and designing targeted initiatives tailored to their needs, we can ensure that our students of all backgrounds can achieve not only core competencies but robust professional actualization.

## Notes

Supplement 1. **eAppendix.** Interview Guide

Supplement 2.
Data Sharing Statement
