# Error in Byline

**DOI:** 10.1001/jamanetworkopen.2024.13418

**Published:** 2024-04-23

In the Original Investigation titled “Challenges Facing First-Generation College Graduates in Medical School: A Qualitative Analysis,”^1^ published December 13, 2023, Mr Parrilla’s name was misspelled in the byline. This article has been corrected.^1^
