# Supplementary material for: Challenges Facing First-Generation College Graduates in Medical School: A Qualitative Analysis
Source: JAMA Netw Open. 2023 Dec 13;6(12):e2347528. doi: 10.1001/jamanetworkopen.2023.47528 (PMC10719755; doi:10.1001/jamanetworkopen.2023.47528)
Supplement: Supplement 1. — eAppendix. Interview Guide [file jamanetwopen-e2347528-s001.pdf]

Supplemental Online Content

Havemann C, Mason HC, Russell RG, et al. Challenges facing first-generation college graduates in medical school: a qualitative analysis. *JAMA Netw Open.* 2023;6(12):e2347528. doi:10.1001/jamanetworkopen.2023.47528

eAppendix. Interview Guide

This supplemental material has been provided by the authors to give readers additional information about their work.

© 2023 Havemann C et al. *JAMA Network Open.*

eAppendix. Interview Guide

Interview Questions from a national, US-based, study of the experiences of first-generation college graduate and/or low-income medical learners. 2021-2022.

1. Tell me a little bit about experiences that shaped your pursuit of a career in medicine.

2. Do you consider yourself as a First-Generation college graduate, from a Low-Income background or Both? In what ways has your FG and/or LI background been an asset during medial training? When has it not?

3. **M4s only**: Can you share a bit about how the application to residency and MATCH process has been for you?

4. What kinds of things take up your time as a medical student that perhaps is not the case for peers who aren't from low-income and/or first-generation college graduate backgrounds?

5. Do you have a story about a time in medical school when you felt like an *outsider, misunderstood, off balance or knocked back etc.*, because of your FG and/or LII background?

6. What aspects of being an FG and/or LI student do you not share with others unless you trust them?
7. How have your perceptions of yourself as a physician in training changed as you've gone through medical school? How has that affected you?

8. Do you feel you belong, are visible, safe and supported and that your school cares about your success as an FG and/or LI medical student?
   a. **If not**, what could faculty, staff and/or your school in general, do to help you feel like you belong are visible, safe and supported?
   b. **If so**, what do faculty and/or your school do that make you and students like you feel you belong, are visible, safe and supported as a student from a FG and/or LI background?
9. What have been the most important supports for you as you've navigated medical school? What supports that are not at your school now, but you wish were?
10. What concrete steps can medical school administrators and faculty take to promote the development of future physicians from backgrounds similar to yours?

© 2023 Havemann C et al. *JAMA Network Open.*

11. Thinking about learners from similar backgrounds like yours - what advice would you give about navigating:
    a. the pre-medical school pathway?
    b. medical school?
12. Do you think your FG and/or LI background will impact your future career path? If so, how so?
13. What will it mean when you can say ‘I am a physician.’?

© 2023 Havemann C et al. *JAMA Network Open.*
