# Supplementary material for: Challenges Facing First-Generation College Graduates in Medical School: A Qualitative Analysis
Source: JAMA Netw Open. 2023 Dec 13;6(12):e2347528. doi: 10.1001/jamanetworkopen.2023.47528 (PMC10719755; doi:10.1001/jamanetworkopen.2023.47528)
Supplement: Supplement 2. — Data Sharing Statement [file jamanetwopen-e2347528-s002.pdf]

Data Sharing Statement

Havemann. Challenges Facing First-Generation College Graduates in Medical School: A Qualitative Analysis. *JAMA Netw Open*. Published December 13, 2023. doi:10.1001/jamanetworkopen.2023.47528

Data
Data available: No

Additional Information
Explanation for why data not available: Privacy of vulnerable student population
